# Assessment of risk factors associated with post-molar gestational trophoblastic neoplasia: a retrospective cohort

**DOI:** 10.61622/rbgo/2024rbgo83

**Published:** 2024-10-23

**Authors:** Silvia Regina Piazzetta, Karin Anspach Hoch, Cristina Laguna Benetti-Pinto, Daniela Angerame Yela

**Affiliations:** 1 Universidade Estadual de Campinas Campinas SP Brazil Universidade Estadual de Campinas, Campinas, SP, Brazil.

**Keywords:** Gestational trophoblastic disease, Gestational trophoblastic neoplasm, Hydatidiform mole, invasive

## Abstract

**Objective:**

Evaluate the risk factors for the development of post-molar gestational trophoblastic neoplasia.

**Methods:**

Retrospective cohort study with 320 women with gestational trophoblastic disease (GTD) followed in a tertiary hospital from January 2005 to January 2020. Data referring to the women's sociodemographic profile, clinical, laboratory and treatment aspects and types of GTD were analyzed.

**Results:**

The mean age of women with the benign form was 26.4±8.6 years and with the malignant forms 26.9±8.5 years (p=0.536). Most women with malignant forms came from regions further away from reference center (p=0.012), had vesicle elimination at the time of diagnosis (p=0.028) and needed more than one uterine evacuation (p<0.001) when compared to the benign forms. There was no difference between laboratory tests in both forms. Being between 30 and 39 years old increased the chance of developing invasive mole by 2.5 (p=0.004; 95%CI:1.3–4.9) and coming from regions far from reference center by 4.01 (p=0.020; CI95%: 1.2-12.9). The women with the highest risk of malignant forms were those with the longest time of become normal on human gonadotrophic hormone (hCG) testing (each week the risk increases 1.3 times; p<0.001, 95%CI: 1.2-1.3).

**Conclusion:**

The prolonged hCG fall curve is the main indicator of an increased chance of GTN. Women from regions further away from reference center have a greater chance of developing malignant forms, probably due to the difficulty in accessing the reference center and, therefore, adequate follow-up that would allow early identification of more serious cases.

## Introduction

Gestational Trophoblastic Disease (GTD) is a term that refers to a heterogeneous group of conditions originating from the abnormal and aberrant proliferation of different types of trophoblastic tissue (syncytiotrophoblast, villous cytotrophoblast and intermediate trophoblast).^(1)^ It is classified into benign forms: Partial hydatidiform mole (PHM) and Complete hydatidiform mole (CHM) and malignant forms called Gestational trophoblastic neoplasia (GTN) which are constituted by invasive mole, choriocarcinoma, epithelioid trophoblastic tumor (ETT) and placental site trophoblastic tumor (PSTT).^(1)^

Persistent elevation or stabilization of human gonadotrophic hormone (hCG) after initial treatment of GTD defines GTN, and histopathological study is not necessary. Histopathology, however, allows a diagnosis of GTN in cases of choriocarcinoma, PSTT or ETT.^(2)^

Gestational trophoblastic neoplasia is a neoplasm with high cure rate when diagnosed early and treated properly.^(3)^ Knowing the factors that increase the risk for the development of post molar GTN allows for a more appropriate and early approach for women. Thus, the aim of this study is to evaluate the risk factors associated with the development of post-molar GTN.

## Methods

This is a retrospective cohort study that evaluated the medical records of 320 women with GTD followed at the GTD outpatient clinic at the State University of Campinas from January 2005 to January 2020. Women with a pathological diagnosis of GTD were included and those who did not have data in the medical records necessary for completing the form were excluded.

The following have been evaluated: age, reference center (women's residence in Campinas and cities up to 50 kilometers from Campinas), race (white and non-white), education (elementary, secondary and higher education), professionally activity (yes or no), obstetric history, gestational age at diagnosis, symptomatology (vaginal bleeding, pelvic pain, hyperemesis, vaginal discharge of vesicles and arterial hypertension), uterine emptying (number of procedures performed), type of GTD (hydatidiform mole, invasive mole, choriocarcinoma, PSTT, ETT), hCG (assessed by chemiluminescence method, expressed in mIU/ml and values below 5mIU/ml are considered normal), hCG time to become negative (weeks), hemoglobin (assessed by blood count - spectrophotometry method, expressed in g/dl), TSH (thyrotrophic hormone evaluated by the electrochemiluminescence method and expressed in mU/l), chest X-ray (chest X-ray – normal or abnormal), uterine volume (measurement of the uterus through transvaginal ultrasound calculated by multiplying the longitudinal, transverse and anteroposterior measurements and an index correction (0.45) measured in cm^3^), metastases, sites of metastases (genital tract, lungs, liver, central nervous system), GTN staging and risk score, hysterectomy (yes or no), chemotherapy (yes or no).

Diagnosis of post-molar GTN followed the FIGO criteria, i.e. rising (10%) hCG levels for three consecutive weeks or plateaued for four weeks, if there is a histologic diagnosis of choriocarcinoma, or when the hCG level remains elevated for 6 months or more from the molar uterine evacuation.^(4-6)^ In 2018, FIGO modified the criteria and a time of more than 6 months for remission was no longer a diagnostic criterion for GTN.^(1)^

GTN staging follows the anatomical staging of the Federation International of Gynecology and Obstetrics (FIGO) published in 2002, which divides GTN into Stage I – disease restricted to the uterus, Stage II – disease in the pelvis, vagina, appendages, broad ligament, Stage III – disease with extension to the lungs, with or without genital involvement and Stage IV – disease with other sites of metastases.^(6)^ The FIGO Prognostic Scoring System factors are the woman's age in years, last pregnancy preceding the GTN (whether molar, abortion or term pregnancy), the interval in months between this pregnancy and the GTN, the pre-treatment hCG value (i.e., hCG at the time of GTN diagnosis, not hCG before molar removal), the size of the largest tumor in centimeters, site and number of metastases, and chemotherapy regimen which was used in case of previous failure. The sum of the scores of each risk factor presented by the woman must be added, with the score ranging from 0 to 26 (Table 1). Women with postmolar GTN with a FIGO score of 0 to 6 are low risk, 7 to 11 high risk, and above 12 (≥12) ultra high risk.^(6)^

**Table 1:** The FIGO Prognostic Scoring System in gestational trophoblastic neoplasia

Variables	0	1	2	4
Age (years)	<40	>40	-	-
Antecedent pregnancy	mole	Abortion	Term	-
Interval from index pregnancy (months)	<4	4-6	7-12	>12
Pre-treatment hCG (UI/L)	<10^3^	10^3^ −10^4^	10^4^-10^5^	>10^5^
Largest tumor size (cm)	-	3-4	≥5	-
Site of metastases	lung	Spleen/kidney	Gastrointestinal tract	Brain/liver
Number of metastases	-	1-4	5-8	>8
Previous chemotherapy	-	-	Single drug	>1 drug

**Source**: FIGO Oncology Committee.^(6)^

To describe the profile of the sample according to the variables under study, the frequency of categorical variables and descriptive statistics of numerical variables with mean and standard deviation values was calculated. For comparison of categorical variables, the chi-square or Fisher's exact tests were used, and for numerical variables, the Mann-Whitney test. To analyze the factors related to malignant neoplasm, Poisson regression analysis was used, simple and multiple (with *Stepwise* variable selection criteria). The significance level adopted for the statistical tests was 5%, that is, p<0.050 and SAS version 9.4 (Cary, NC, USA) was used for the statistical analysis. The sample power calculation was carried out with the objective of evaluating the risk factors for developing gestational trophoblastic neoplasia considering the main risk factor being the average time for hCG normalization among malignant forms, with estimates obtained from the current sample, fixing the level of significance at 5%. Based on the results, a power of 99.9% was verified.

This study was approved by the Research Ethics Committee of the institution 4.666.443 (number: 43974021.0.0000.5404).

## Results

**Figure 1:**
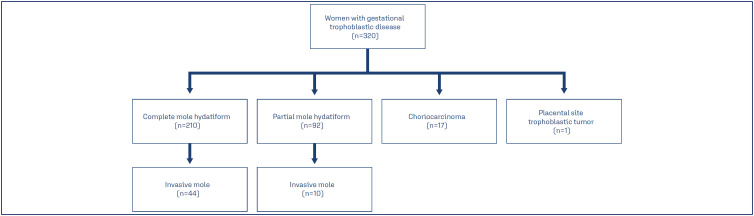
Distribution for the women with gestational trophoblastic disease

The median of age of women was 25.0, with the youngest age being 13.0 years old and the oldest age being 53.0 years old. Gestational trophoblastic disease types were 52.0% complete mole, 25.5% partial mole, 16.8% invasive mole, 5.3% choriocarcinoma and 0.1% PSTT. Of the post-molar GTN, 81.5% was preceded by complete mole and 18.5% by partial mole (Figure 1). Of the cases of post-molar GTN, 79.6% were in stage I, 1.8% in stage II, 14.8% in stage III and 3.7% in stage IV; 96.5% had a risk score less than or equal to 6 and 3.5% had a risk score greater than 6. Regarding the treatment, 6 women underwent hysterectomy and 49 women underwent chemotherapy, with the most commonly used regimen being monochemotherapy with methotrexate (Table 2).

**Table 2:** Clinical characteristics of women with gestational trophoblastic disease (n=320)

Variables		n(%)	mean±SD	Median
Age (Years)			26.5±8.5	25.00
Previous pregnancies				
	1	100(31.0)		
	≥2	85(26.5)		
Previous C-section		76(23.7)		
Previous mole		10 (3.1)		
BMI (Kg/m2)			24.7±5.0	
White		77.7		
GTD types				
	Complete mole	166(52.1)		
	Partial mole	82(25.5)		
	Invasive mole	54(16.8)		
	Choriocarcinoma	17(5.3)		
	PSTT	1 (0.3)		
Staging				
	I	43(79.6)		
	II	1(1.8)		
	III	8(14.8)		
	IV	2(3.7)		
Risk Score				
	≤ 6	52 (96.5)		
	>6	2 (3.5)		

BMI - body mass index, GTD - gestational trophoblastic disease, PSTT - Placental site trophoblastic tumor, SD - standard deviation

The average age of women with the regression after uterine evacuation was 26.4±8.6 years and with the progression to GTN 26.9±8.5 years (p= 0.536) although most women with GTN were in the age range from 30 to 39 years and those without GTN were younger (p=0.006). In both groups, most women were white (p=0.341), with a partner (p=0.429), with primary education (p=0.350), with professional occupation (p=0.786) and with an average of 2 previous pregnancies (p=0.530). Most women with GTN came from regions further away from reference center (p=0.012) (Table 3).

**Table 3:** Sociodemographic characteristics of women with and without gestational trophoblastic neoplasia (n=320)

Variables		GTN (n=54) mean±SD / n(%)	Without GTN (n=266) mean±SD / n(%)	p-value
Age (years)		26.9±8.5	26.4±8.6	0.536*
Age gap (years)				0.006**
	<20	13(24.0)	61(22.9)	
	20 a 29	16(29.6)	125(47.0)	
	30-39	22(40.7)	53(19.9)	
	≥40	3(5.5)	27(10.2)	
BMI (Kg/m2)		25.7±6.1	24.4±4.6	0.189*
White		42(77.7)	190(71.4)	0.341**
With partner #		35(66.0)	183(71.4)	0.429**
Education #				0.350**
	Elementary	28(50.1)	110(63.9)	
	Secondary	13(36.1)	40(23.2)	
	Higher Education	5(13.8)	22(12.7)	
Place of residence				0.012***
	Reference center	41(75.9)	233(87.9)	
	Out reference center	13(24.1)	32(12.1)	
Professionally activity (yes)		30(55.1)	116(43.8)	0.786***
Number of pregnancies		2.0±1.2	2.0±1.2	0.530*

GTN - gestational trophoblastic neoplasia; BMI - body mass index; SD - standard deviation;

* Mann-Whitney Test;

** Chi-square Test;

*** Fisher test;

# Missing data

There was no difference between laboratory tests in both groups. Most women with progression to GTN came from regions further away from reference center (p=0.012), had vesicle elimination at the time of diagnosis (p=0.028) and needed more than one uterine evacuation (p<0.001) when compared to women with regression after uterine evacuation. The time for to hCG level to become normal was longer in women with GTN (p<0.001) (Table 4).

**Table 4:** Clinical and laboratorial characteristics of women with and without gestational trophoblastic neoplasia (n=320)

Variables		GTN (n=54) mean±SD / n(%)	Without GTN (n=266) mean±SD /n(%)	p-value
Gestational age (weeks)		11.2±3.1	12.0±3.5	0.160*
Symptoms				
	Pain	29(53.72)	93(34.9)	0.010**
	Bleeding	44(81.4)	195(73.3)	0.200**
	Vesicle elimination	9(16.6)	18(6.7)	0.028***
	Hyperemesis	(8)14.8	42(15.7)	0.850**
	Arterial hypertension	4(7.4)	9(3.3)	0.240***
Uterine evacuation				0.001***
	1	28(51.8)	200(75.4)	
	≥2	26(48.2)	66(24.6)	
Initial hCG (mIU/ml)		324,436±381,738	247,409±344,400	0.220*
Time to become normal hCG (weeks)		5.6±2.8	2.6±2.4	<0.001*
Haemoglobin (g/dl)		11.7±1.9	12.0±1.5	0.370*
TSH (mU/l)		1.4±1.0	1.7±1.5	0.490*
Uterine volume (cm^3^)		400.8±335.7	408.2±436.8	0.930*
Altered chest x-ray		13(24.0)	16(6.1)	<0.001***

GTN - gestational trophoblastic neoplasia; SD - standard deviation; hCG - human gonadotrophic hormone; TSH - Thyroid stimulant hormone;

* Mann-Whitney Test;

** Chi-square Test;

*** Fisher Test;

# missing data

Being between 30 and 39 years old increased the chance of developing invasive mole by 2.5 (p=0.004; 95%CI:1.3–4.9) and coming from regions far from reference center by 4.0 (p=0.020; CI95%: 1.2 - 12.9). The women with the highest chance of GTN were those with the longest time to normalization hCG (every week the risk increases 1.3 times; p<0.001, 95%CI: 1.2-1.3) (Table 5).

**Table 5:** Factors correlated with the development of gestational trophoblastic neoplasia (n=320)

Variables	p-value	HR (IC95%)	p-value	Adjusted HR* (IC95%)
Age (years)				
<20	0.240	1.5(0.7-3.2)		
20 a 29 (ref)	-	-		
30-39	0.004	2.5(1.3-4.9)		
≥40	0.840	0.8(0.2-3.0)		
Reference center				
In (ref)	-	-		
Out	0.020	4.0(1.2-12.9)		
Race				
White (ref)	-	-		
Non-white	0.380	0.7(0.4-1.4)		
Initial hCG (mIU/ml)	0.260	1.0(0.9-1.0)		
Time to become normal hCG (weeks)	<0.001	1.1(1.0 −1.1)	<0.001	1.2(1.2-1.3)
Haemoglobin (g/dl)	0.190	0.9(0.7-1.0)		
TSH (um/l)	0.290	0.8(0.6-1.1)		
Uterine volume (cm^3^)	0.900	1.0(0.9-1.0)		
Chest x-ray				
Normal (ref)	-	-		
Altered chest x-ray	<0.001	3.1(1.6-5.8)		

HR - Hazard Ratio; CI 95% - 95% confidence interval; ref - reference level; hCG - human chorionic gonadotrophin; TSH - thyroid stimulating hormone

## Discussion

This study showed 54 cases of post-molar GTN. Of the GTN cases, most were diagnosed in stage I and with a low risk score (below ≤ 6). Women with GTN had a higher age group than women without GTN and lived further away from reference center. They also had more symptoms (pain and elimination of vesicles) and the longer time to negativity hCG. The factors associated with a greater chance of developing GTN were being aged between 30 and 39 years, living outside reference center and delay in the time to normalization hCG.

Most cases of post molar GTN were due to complete mole (81.5%) and the percentage of choriocarcinoma was 5%. These numbers are similar to other studies in the literature.^(7,8)^

We observed that although there was no difference in the mean overall age between the groups, most women with GTN were in the age range of 30 to 39 years and those without GTN were younger (p=0.006), data that are very similar to that found in study carried out in Porto Alegre in the late 1990s, which observed a higher incidence of hydatidiform mole in the population under 30 years of age, but greater progression to neoplasia in the range between 31-35 years and after 40 years.^(9)^ International studies show an even greater risk of progression to GTN in women over 40 years of age^(10,11)^ with some protocols recommending the possibility of hysterectomy for this group of women.

In our study, women with GTN were older (more than 30 years) than women without GTN, although age is not a factor that increases the chance of developing GTN. A study in the literature also showed no association between age and GTN,^(7)^ although other studies show an association between advanced age and increased risk of GTN.^(12,13)^ These discrepancies may be related to study characteristics such as the type of previous molar pregnancies, selection bias and, perhaps, the sociodemographic characteristics of the studied population.

In our study, we found no difference between the ethnicity of women with and without GTN, and ethnicity was not related to a greater chance of developing GTN. In the literature a study showed that white women with complete mole had a greater chance of developing GTN than the others. In that study, when evaluating white women with a partial mole, this did not show an association with the risk for GTN. However, there were no significant differences between racial/ethnic groups in terms of symptom presentation, gestational age at diagnosis, or hCG levels preevacuation for women.^(14)^

The adaptation of post molar follow-up protocols requires further studies so that they can be adjusted according to the type of molar disease and the falling hCG curves. The individualization of care with selection of higher risk cases and shortening of the period of follow up could turn out to be more cost effective and provide greater adherence. Mainly when we consider the geographic and economic barrier existing in Brazil. Women from regions further away from reference center are more likely to evolve into malignant forms, probably due to the difficulty in accessing the reference center.

Our study showed that being far from the reference center increases the risk of developing neoplasia. A recent study that assessed the distance between a woman's residence and the reference center showed that greater distance increases the risk of unfavorable outcomes and death.^(15)^ This result of our study, women who live in places further away from the reference center having more GTN may be related to a referral bias since places that are further away and with fewer resources will only refer cases with a worse prognosis or in need of more specific follow-up, such as oncology follow-up.

In our study, longer hCG normalization time was the factor with the greatest association with the development of post-molar GTN. We observed that women whose hCG takes longer to normalize are those who are more likely to develop GTN, as shown by several studies in the literature. In our study, women who developed GTN had a mean hCG normalization of 6 weeks after chemotherapy, while those who did not develop GTN had a spontaneous regression within 3 weeks. The longer normalization of women with GTN was due to the need for chemotherapy to normalize hCG. One study discusses this as the main marker for progression to GTN, although it cites other factors in the literature, such as the presence of theca lutein cysts, large uterine height for gestational age and history of miscarriage.^(16)^ Other studies also indicate the hCG curve as the main factor for post-molar GTN.^(7,17,18)^ Another study shows that hCG≥ 20,000 IU/L four weeks after uterine emptying increases the chance of developing GTN.^(19)^ The literature shows that an inadequate drop in hCG values after 14 days may predict a greater risk for the development of malignant forms.^(20)^

This study has several limitations. It is a retrospective study from a single institution and therefore exposed to inherent references and historical biases, and the study includes a relatively small number of patients. Another limitation is Grimes’ Gray Zone to interpret the results.^(21)^

In a country with wide geographic dimensions and unequal access to health, as in Brazil, alternative strategies that facilitate post-molar follow-up have been the focus of the studies. Proposed urinary hCG concentrations as markers and telemedicine assistance are examples of methods that have been analyzed as a way to minimize distances and improve adherence. Knowledge of risk factors associated with progression to GTN makes it possible to adjust the follow-up period in different scenarios.

## Conclusion

The prolonged hCG fall curve is the main indicator of an increased chance of GTN. Women from regions further away from reference center have a greater chance of evolving to malignant forms, probably due to the difficulty in accessing the reference center and, therefore, adequate follow-up that would allow an early identification of more serious cases.
